# Inflammatory Wnt5A signalling pathways affecting barrier function of human vascular endothelial cells

**DOI:** 10.1186/s12950-017-0163-6

**Published:** 2017-07-13

**Authors:** Tom Skaria, Gabriele Schoedon

**Affiliations:** Inflammation Research Unit, Division of Internal Medicine, University Hospital Zürich, Rämistrasse 100, CH-8091 Zürich, Switzerland

**Keywords:** Inflammation, Wnt5A, Endothelial barrier function, Ryk, IL-4, Wnt inhibitory factor-1

## Abstract

Wnt5A is a chemokine secreted by inflammatory-activated human macrophages that sustains their inflammatory response in an autocrine manner. High levels of Wnt5A are found in sera of patients with sepsis and septic shock.

Here, we comment on recently reported Wnt5A signalling pathways in human vascular endothelial cells (VEC). In human VEC, Wnt5A regulates cytoskeleton remodelling and barrier function through Ryk receptor and Rho-associated protein serine/threonine kinase, targeting LIMK2 and CFL1 involved in actin polymerisation. Wnt5A/Ryk signalling in VEC can be antagonised by the naturally occurring Wnt inhibitory factor (WIF)-1 (WIF1).

Therapeutic targeting of this mechanism may reduce vascular leakage and edema in severe systemic inflammation and therefore should be subject of further investigations.

Vascular leakage caused by endothelial barrier dysfunction is a critical feature of severe systemic inflammatory diseases like sepsis, or allergic responses and autoimmune disorders. Wnt5A has been identified as a chemokine secreted by Toll-like receptor (TLR)-activated human macrophages that is crucially involved in the autocrine modulation of their inflammatory response [1, 2]. Here, we comment on the novel finding of a Wnt5A/Ryk signalling mechanism regulating cytoskeleton remodelling and barrier function in adult human immunocompetent vascular endothelial cells (VEC).

In our recent study employing differential gene expression profiling of VEC treated with the potent Th2 cytokine IL-4, Wnt5A was surprisingly found among the top upregulated genes [3]. The top most significant pathways regulated by IL-4 in VEC were those containing genes associated with cytoskeleton remodelling, for which Wnt5A might be a potential ligand. The genes clustered in these pathways were *LIMK2,* a serine/threonine/tyrosine kinase phosphorylated by the activation of Rho-associated protein serine/threonine kinase (ROCK), and the actin depolymerization factor *CFL1* [3]*.* A separate study employing whole genome transcriptome profiling of paracrine Wnt5A treatment in primary VEC verified that the same cytoskeleton remodelling pathway involving *LIMK2* and *CFL1* is the cellular process most significantly regulated by Wnt5A in VEC [4]*.* Phosphorylated LIMK2 phosphorylates, and thereby inactivates CFL1 leading to enhanced actin polymerization. In response to IL-4 and Wnt5A treatments, LIMK2 and CFL1 were phosphorylated, and actin stress fiber formation was enhanced [3, 4]. A crucial involvement of autocrine Wnt5A in IL-4 induced cytoskeleton remodelling process was revealed by the finding that actin stress fiber formation in IL-4-treated VEC was notably decreased in the presence of the Ryk receptor specific Wnt antagonist, Wnt inhibitory factor (WIF)-1 (WIF1). Barrier dysfunction of IL-4- treated VEC monolayers was found significantly suppressed by silencing Wnt5A [3]. Likewise, silencing Ryk expression prevented Wnt5A-mediated endothelial barrier dysfunction [4]. It has been further shown that WIF1 specifically prevents Wnt5A-mediated LIMK2/CFL1 phosphorylation and adherens junction disruption [5]. Collectively, these data reveal that the Wnt5A/Ryk pathway regulates actin cytoskeleton remodelling and monolayer barrier function of human VEC through ROCK/LIMK/CFL signalling. The transcription factors and the molecular mechanism mediating Wnt5A induction in response to IL-4 treatment in VEC still remain unclear and warrant further studies.

Importantly, it was further shown that Wnt5A does not regulate the expression of proinflammatory cytokines or intercellular adhesion molecules in human VEC [4], in contrast to the Frizzled (Fzd)-5 receptor mediated Wnt5A signalling in macrophages [1, 2]. Wnt5A can trigger multiple signalling pathways depending on the cellular milieu, the availability of receptors, and naturally occurring Wnt antagonists [6]. Therefore, the ability of Wnt5A to engage two distinct receptors and signalling pathways in macrophages and VEC is not surprising.

Taken together, recent studies mentioned [3–5] strongly indicate that Wnt5A/Ryk signalling mediates cytoskeleton remodelling and barrier dysfunction in human VEC, and these processes are critically modulated by the endogenous protein WIF1 [5]. Previous studies report elevated levels of Wnt5A in sera of patients with severe sepsis and septic shock [2], that is corroborated by the finding of strongly induced synthesis and secretion of Wnt5A by TLR-activated macrophages. This along with the present finding of highly induced Wnt5A expression in IL-4-activated VEC prompt further studies to investigate if Wnt5A contributes to vascular leakage and edema formation in severe systemic inflammatory diseases like sepsis/septic shock, and in IL-4 driven allergic inflammation. Investigations are also needed to unravel the intriguing finding of Wnt5A being induced by two different inflammatory pathways that are evolutionary highly conserved, TLR-dependent and IL-4 dependent signalling. Targeting paracrine, as well as autocrine Wnt5A signalling by WIF1 may open novel therapeutic perspectives for the treatment of capillary leakage in systemic inflammatory conditions such as severe inflammation and sepsis, as well as allergic and autoimmune diseases.

## Abbreviations

Fzd: Frizzled
ROCK: Rho-associated protein serine/threonine kinase
TLR: Toll-like receptor
VEC: Vascular endothelial cells
WIF1: Wnt inhibitory factor-1
